# Severe Dysphagia After Inferior Alveolar Nerve Block Preceded By Cervical Botolinum Toxin Injection: A Case Report

**DOI:** 10.5812/ircmj.7427

**Published:** 2013-07-05

**Authors:** Gholamali Shahidi, Arash Poorsattar Bejeh Mir, Ramin Khatib Shahidi, Pouya Balmeh

**Affiliations:** 1 Division of Movement Disorder, Department of Neurology, Rasool-e Akram Hospital, Tehran University of Medical Sciences, Tehran, IR Iran; 2 Dentistry School, Babol University of Medical Sciences, Babol, IR Iran

**Keywords:** Deglutition Disorders, Anesthesia, Local, Botulinum Toxins, Cervical Dystonia, Side Effect

## Abstract

A 39-year-old Iranian female patient who was a known case of primary cervical dystonia since 10 years ago presented to a private office for Dysport injection. The patients experienced severe dysphagia after inferior alveolar nerve block which was preceded by cervical botolinum toxin injection. A possible synergistic effect of botolinum toxin and lidocaine to produce severe dysphagia is presented in this case report.

## 1. Introduction

Idea for possible therapeutic usage of botolinum toxin (BTX) dates back to 18th and 19th century proposed first by Justinus Kerner (1786 - 1862). He called this toxin “sausage poison” (i.e. botulus means sausage in Latin) (1). Since 1895 and explanation of BTX mechanism of action, a variety of beneficial effects treating various diseases are introduced. BTX decreases muscular activity by reducing the release of presynaptic neurotransmitter acetylcholine on the cholinergic nerve terminals; hence confer paralysis at the level of neuromuscular junction (1). Four main types of BTX for cosmetic and therapeutic purposes are currently being manufactured: Onabotulinum toxin A (Botox), Abobotulinum toxin A (Dysport), Incobotulinum toxin A (Xeomin) and Rimatoxin B (Myobloc). Indications for BTX therapy in head and neck region may be addressed to the treatment of hemifacial spasm, headache, myokimia, myoclonus of tensor veli palatini, hypersialorrhea, blepharospasm, tics, strabismus, cervical dystonia, temporomandibular joint disorders related to the muscular spasticity and hyperkinetic lines in forehead and around the eyes (1).

Up to one-third of patients may suffer any of BTX injection complications (2). Neck weakness, pain at the site of injection and dysphagia are among the most common complaints. Most reports of dysphagia are mild to moderate and more severe forms are not common (1). Inferior alveolar nerve block (IANB) is commonly used to give anesthesia over the soft and had tissue of the corresponding quadrant with the exception of buccal area of molars that receive sensory branches form the buccal nerve. Inadvertent vascular injection may be best avoided by proper direction and twice aspiration. Severe neurologic complications may occur after the incidental vascular injections and even total body hemiparesis may be preceded by circulation of the anesthetic to the brain. Rarely, inadvertent insult to prevertebral space and stellate ganglion during IANB may lead to Horner syndrome (3). A possible synergistic effect of botolinum toxin and lidocaine to produce severe dysphagia is discussed in this case report.

## 2. Case Presentation

A 39-year-old Iranian female patient who was a known case of primary cervical dystonia since 10 years ago presented to a private office for Dysport injection. She was otherwise a healthy person with no other systemic problem. She took 2 mg trihexyphenidyl and 1 mg clonazepam daily. The patient received her first BTX injection about six years ago, although details regarding the amounts and sites of injections were not available. She abandoned Dysport injection due to the finical problems. On physical examination, she has right torticollis, left laterocollis, head shaking and shoulder elevation with no other clinical problem. Her second injection was performed by the following dosages and sites: 200 U at right splenius capitis, 100 U at left sternocleidomastoid, 100 U at left scalen, 50 U at left splenius capitis and 50 U at left trapezius muscle.

On the 2nd following day, she sought dental care for mandibular molar root canal therapy. Inferior alveolar nerve block was performed by local administration of 1.8cc containing lidocaine 2% and 1:80,000 epinephrine. Dental procedure was uneventful and she was discharged with routine analgesic prescription. The patient suffered from difficulty with swallowing solid foods in a progressive manner that she was unable to drink 24 hours after IANB. Thereafter, she was admitted to the hospital for severe dysphagia. Supportive care initiated and she was kept NPO and received fluids by gastric gavage for five consecutive days. On hospital examination, she was mildly dysphonic; she had mild dystonia and cervical weakness and was totally unable to initiate to swallow when a spoon of water was distilled in her mouth. Her neurologic examination except weakness of the bulbar musculature was normal otherwise. There was no electrolyte disturbance and no further imaging study accomplished. Her dysphagia was gradually improved and she switched to soft liquid at 5th day of admission and the patient was discharged at the 7th day. She was followed two and 25 days after hospital discharge. On her last visit, dysphagia was completely improved; however she had a mild cervical dystonia.

## 3. Conclusions

A case of sever dysphagia following inferior alveolar nerve block and botolinum toxin injection at cervical region is presented. Our patient experienced progressive dysphagia with difficulty swallowing solid foods that then liquids. To our knowledge, it is the first report that introduces possible synergistic effect of lidocaine and BTX lead to severe dysphagia with the both oropharyngeal and esophageal transitions affected. Reviewing literature, there are reports of severe dysphagia after therapy with Botox and Dysport, although severe form does not occur on a frequent basis (4). Overall, higher rates of complications are reported when women or sternocleidomastoid are injected. Dysphagia is the most remarkable complication reported after BTX injection to treat cervical dystonia. As high as one-third of patients experienced dysphagia receiving their first injection, especially when sternocleidomastoid was targeted and dose exceeded 100 U. Repetitive injection with accumulation of toxin increases the chance for such distant side effects (4). Also, a bolus single site and high dose injection increases the risk for blood-stream spreading of the toxin as a consequent of attenuated in situ bindings to the cholinergic terminals.

It is unlikely that dysphagia in the case presented was related to systemic infusion of anesthetic or deep penetration into the pharyngeal spaces and stellate ganglion blockade. Since cranial nerves were intact (e.g., gag reflex was present, uvula was not deviated and shoulder elevation, facial expression and pupil size were normal) direct invasion of carotid sheath was not presumed to be the case. Briefly, possible mechanisms for the explanation of dysphagia preceding IANB and BTX therapy are as the followings: 1-diffusion of either lidocaine or BTX to the adjacent carotid sheath and paralysis of within IX-XIth cranial nerves, 2- direct diffusion to the adjacent muscles and induction of paralysis at motor-end-plate or neuromuscular junction level with possible synergistic effect of lidocaine and BTX. Botolinum toxin may diffuse 30 - 45mm around the site of injection, although further spreading depends on anatomic features of the location, dosage and probably the type of botolinum toxin and its preparation (4). An explanation to combined deficient oropharyngeal and esophageal phases may be the paralysis of corresponding muscles to both phases. As mandibular branch of 5th cranial nerve enters the inferior alveolar nerve canal, it gives rise to motor branch named mylohyiod nerve (MHN) just 15 mm above the canal. This nerve innervates anterior belly of digastric muscle and myelohyiod muscle. These muscles are important for initiating of the oral phase of swallowing by backward rotation of mandible and elevation of the floor of the mouth. In addition, a sensory motor to lingual nerve is described that may adversely affect the eating when get paralysed (5). Since documentation of this aberrant branch needs surgical exploration, we are uncertain of the presence of this communication. It is noteworthy to mention that proper functioning mylohyiod muscle is essential for phonation, inspiration, chewing and swallowing. Mild dysphonia in this patient probably was the consequence of affected mylohyiod muscles and direct diffusion of toxin into the laryngeal supporting muscles or even paralysis of recurrent laryngeal nerve, as previously described.

In this certain case, based on recent history of BTX injection and IANB and no significant past medical history suggestive of mechanical causes for dysphagia, most probable reason for such acute and severe form of dysphagia was the injections. Functional dysphagia could be appropriately worked up by video fluoroscopy or Flexible Endoscopic Evaluation of Swallowing (FEES). These diagnostic tools were not available at the hospital where the patient was admitted. She was uncooperative to perform a barium swallow study; hence no additional imaging study was carried out for her. Proposed synergistic effect of botolinum toxin and lidocaine needs future in-vitro and in-vivo approval investigations. There are evidences of beneficial features of lidocaine to alleviate pain and improve dystonia in patients with secondary dystonia due to parkinson disease or complex regional pain syndrome (CRPS) previously known as reflex sympathetic dystrophy syndrome (RSDS) (6, 7). Probable involvement of afferent sensory fibers in the etiology of dystonia raises this interesting therapeutic target to treat dystonia (6). Importantly, combination therapy, especially in cervical region, should be performed with great care regarding the serious side effect occurred in our patient. In order to lessen serious side effects, the frequency and amount of injections should be planned at the least possible times and dosage. We believe that Botulinum toxin produces some degree of weakness in several neck muscles and affect swallowing most often not sever enough to produce clinical symptoms. The local anesthetic effects on already weakened muscles have produced severe but transient dysphagia. It is wise to postpone non-emergent procedures such as dental care with lidocaine administration that have serious common side effect of dysphagia with BTX injection, especially when injected in the cervical region and more than 100u.
